# Supplementary material for: Comparative genomics provides new insights into the diversity, physiology, and sexuality of the only industrially exploited tremellomycete: Phaffia rhodozyma
Source: BMC Genomics. 2016 Nov 9;17:901. doi: 10.1186/s12864-016-3244-7 (PMC5103461; doi:10.1186/s12864-016-3244-7)
Supplement: Additional file 6: — List of orphan genes with links to PFAM (related to Additional file 1: Table S1). (ZIP 1428 kb) [file 12864_2016_3244_MOESM6_ESM.zip › PFAM_UNIQUE/G05118_P.html]

#                                                                            --- full sequence --- -------------- this domain -------------   hmm coord   ali coord   env coord
  
# target name        accession   tlen query name           accession   qlen   E-value  score  bias   #  of  c-Evalue  i-Evalue  score  bias  from    to  from    to  from    to  acc description of target
  
#------------------- ---------- ----- -------------------- ---------- ----- --------- ------ ----- --- --- --------- --------- ------ ----- ----- ----- ----- ----- ----- ----- ---- ---------------------
  
G05118\_P             -            212 ATP-synt\_E           PF05680.7     90       2.4    6.9  17.0   1   2     0.056        56    2.5   0.9    25    75    28    78    22    93 0.46 - ATP synthase E chain  
G05118\_P             -            212 ATP-synt\_E           PF05680.7     90       2.4    6.9  17.0   2   2   0.00048      0.48    9.1   9.2    36    82   111   156   106   187 0.69 - ATP synthase E chain  
G05118\_P             -            212 AvrE                 PF11725.3   1774       1.1    4.9  11.7   1   1     0.001       1.2    4.7  11.7    85   195    31   162     6   205 0.56 - Pathogenicity factor  
G05118\_P             -            212 BLVR                 PF06375.6    154         6    5.3  25.2   1   2       1.3   5.2e+02   -1.0   3.9    69    87    42    59    14    79 0.51 - Bovine leukaemia virus receptor (BLVR)  
G05118\_P             -            212 BLVR                 PF06375.6    154         6    5.3  25.2   2   2   0.00012     0.046   12.2  14.5    28   112    69   157    62   183 0.57 - Bovine leukaemia virus receptor (BLVR)  
G05118\_P             -            212 BTV\_NS2              PF04514.7    363      0.34    8.4  16.2   1   1   0.00085      0.46    8.0  16.2   132   270     9   155     2   169 0.63 - Bluetongue virus non-structural protein NS2  
G05118\_P             -            212 Dicty\_REP            PF05086.7    911       4.6    3.5  13.9   1   1      0.01         6    3.1  13.9   239   344    44   151    16   162 0.52 - Dictyostelium (Slime Mold) REP protein  
G05118\_P             -            212 Draxin               PF15550.1    324      0.02   13.2   8.3   1   1   2.4e-05     0.024   13.0   8.3    21   146    42   163    23   197 0.62 - Draxin  
G05118\_P             -            212 DUF2486              PF10667.4    246       1.9    7.7  10.9   1   1   0.00072       2.2    7.4  10.9    24   134    42   155    14   196 0.57 - Protein of unknown function (DUF2486)  
G05118\_P             -            212 DUF3801              PF12687.2    204       2.7    6.2  11.4   1   1    0.0048       3.6    5.8  11.4   110   204    30   143     4   188 0.66 - Protein of unknown function (DUF3801)  
G05118\_P             -            212 DUF4588              PF15251.1    233      0.72    8.5   5.8   1   1   0.00078      0.93    8.1   5.8   107   220    34   148    21   188 0.69 - Domain of unknown function (DUF4588)  
G05118\_P             -            212 DUF755               PF05501.6    123       5.5    5.7   7.7   1   2       1.2   8.7e+02   -1.4   0.3    72    85    46    59    31    75 0.53 - Domain of unknown function (DUF755)  
G05118\_P             -            212 DUF755               PF05501.6    123       5.5    5.7   7.7   2   2   0.00062      0.46    9.2   2.3    69   111   110   154   103   161 0.53 - Domain of unknown function (DUF755)  
G05118\_P             -            212 FAM196               PF15265.1    514       9.2    4.0  11.6   1   1      0.03        11    3.7  11.6   141   280    29   167     7   200 0.46 - FAM196 family  
G05118\_P             -            212 FLO\_LFY              PF01698.11   387       1.1    6.7  13.5   1   1    0.0028       1.4    6.3  13.5   121   217    50   156    34   180 0.51 - Floricaula / Leafy protein  
G05118\_P             -            212 Herpes\_DNAp\_acc      PF04929.7    381       9.3    3.7  11.6   1   1    0.0074        15    3.1  11.6   295   363    48   146    14   157 0.54 - Herpes DNA replication accessory factor  
G05118\_P             -            212 Hex\_IIIa             PF02455.11   491       9.7    3.4  10.0   1   1     0.015        11    3.2  10.0   352   466    38   151    11   180 0.44 - Hexon-associated protein (IIIa)  
G05118\_P             -            212 HSP70                PF00012.15   602       6.4    3.2  10.6   1   1     0.017       7.9    2.9  10.6   511   559    52   157    20   190 0.62 - Hsp70 protein  
G05118\_P             -            212 LAP1C                PF05609.7    465       2.1    5.5  13.2   1   1    0.0012       2.3    5.4  13.2    17   139    29   153    13   204 0.58 - Lamina-associated polypeptide 1C (LAP1C)  
G05118\_P             -            212 MIP-T3               PF10243.4    539       1.1    6.3  24.1   1   1     0.011       1.4    5.9  24.1   136   243    38   161    11   206 0.48 - Microtubule-binding protein MIP-T3  
G05118\_P             -            212 Mitofilin            PF09731.4    582       6.5    3.9  20.0   1   1     0.073       8.7    3.5  20.0    66   173    33   175     7   203 0.42 - Mitochondrial inner membrane protein  
G05118\_P             -            212 Mito\_fiss\_reg        PF05308.6    253       1.1    7.3   7.3   1   1    0.0054       1.8    6.5   7.3   152   234    59   141    25   154 0.63 - Mitochondrial fission regulator  
G05118\_P             -            212 NST1                 PF13945.1    190     0.066   11.9  10.6   1   2     0.089        67    2.1   0.5    22    52    43    73    26    95 0.45 - Salt tolerance down-regulator  
G05118\_P             -            212 NST1                 PF13945.1    190     0.066   11.9  10.6   2   2   2.1e-05     0.015   14.0   3.9     8    57   112   161   105   197 0.58 - Salt tolerance down-regulator  
G05118\_P             -            212 ORC6                 PF05460.8    353       5.6    4.6  11.0   1   1     0.025       6.9    4.3  11.0   129   213    31   148     2   187 0.68 - Origin recognition complex subunit 6 (ORC6)  
G05118\_P             -            212 PcfK                 PF14058.1    140       2.1    7.2  18.9   1   2    0.0056       8.4    5.2   2.3    72   114    44    85    17    93 0.58 - PcfK-like protein  
G05118\_P             -            212 PcfK                 PF14058.1    140       2.1    7.2  18.9   2   2    0.0011       1.6    7.5   9.0    89   128   107   146    98   158 0.80 - PcfK-like protein  
G05118\_P             -            212 RNA\_polI\_A34         PF08208.6    198      0.47    8.8  23.1   1   2     0.064        29    2.9   4.0   149   181    41    61    10    91 0.54 - DNA-directed RNA polymerase I subunit RPA34.5  
G05118\_P             -            212 RNA\_polI\_A34         PF08208.6    198      0.47    8.8  23.1   2   2    0.0001     0.047   12.0  11.2   144   187   109   150    97   183 0.64 - DNA-directed RNA polymerase I subunit RPA34.5  
G05118\_P             -            212 Rrp15p               PF07890.7    130       8.9    5.0  12.4   1   2     0.038        23    3.7   0.5    28    63    42    77    28    97 0.63 - Rrp15p  
G05118\_P             -            212 Rrp15p               PF07890.7    130       8.9    5.0  12.4   2   2    0.0091       5.5    5.7   5.6    34    64   115   144   102   194 0.55 - Rrp15p  
G05118\_P             -            212 TFIIF\_alpha          PF05793.7    528       3.8    4.4  43.4   1   2      0.35        62    0.4   9.0   314   354    38    77    14    95 0.67 - Transcription initiation factor IIF, alpha subunit (TFIIF-alpha)  
G05118\_P             -            212 TFIIF\_alpha          PF05793.7    528       3.8    4.4  43.4   2   2   0.00098      0.17    8.8  28.1   310   392   107   183    82   200 0.58 - Transcription initiation factor IIF, alpha subunit (TFIIF-alpha)  
G05118\_P             -            212 TORC\_M               PF12885.2    150       9.3    4.7  10.9   1   2     0.012        71    1.8   0.7    17    71    17    72     8    86 0.56 - Transducer of regulated CREB activity middle domain  
G05118\_P             -            212 TORC\_M               PF12885.2    150       9.3    4.7  10.9   2   2   0.00026       1.6    7.2   3.7    12    67    93   149    89   157 0.74 - Transducer of regulated CREB activity middle domain  
G05118\_P             -            212 Trypan\_glycop\_C      PF10659.4     97       7.9    5.5  17.1   1   2    0.0041        24    4.0   2.4    30    51    46    67    25    94 0.43 - Trypanosome variant surface glycoprotein C-terminal domain  
G05118\_P             -            212 Trypan\_glycop\_C      PF10659.4     97       7.9    5.5  17.1   2   2   0.00043       2.6    7.1   7.0    34    65   115   148   104   172 0.49 - Trypanosome variant surface glycoprotein C-terminal domain  
G05118\_P             -            212 U3\_snoRNA\_assoc      PF08297.6     88       2.4    7.2  14.7   1   2     0.013        16    4.5   0.4    18    43    37    62    12    73 0.69 - U3 snoRNA associated  
G05118\_P             -            212 U3\_snoRNA\_assoc      PF08297.6     88       2.4    7.2  14.7   2   2    0.0044       5.3    6.0   8.2    14    50   109   145    97   149 0.72 - U3 snoRNA associated  
G05118\_P             -            212 Utp14                PF04615.8    735      0.65    6.7  22.4   1   1    0.0084      0.78    6.5  22.4   424   531    38   162    14   204 0.41 - Utp14 protein  
G05118\_P             -            212 Vfa1                 PF08432.5    182      0.48    9.1  24.4   1   2      0.12        29    3.3   3.9    96   124    43    71     9    92 0.44 - AAA-ATPase Vps4-associated protein 1  
G05118\_P             -            212 Vfa1                 PF08432.5    182      0.48    9.1  24.4   2   2   0.00027     0.065   11.9  12.6    60   114   109   161    97   192 0.68 - AAA-ATPase Vps4-associated protein 1
